# Iloprost modulates the immune response in systemic sclerosis

**DOI:** 10.1186/1471-2172-11-62

**Published:** 2010-12-15

**Authors:** Patrizia D'Amelio, Maria A Cristofaro, Lucia D'Amico, Luciana Veneziano, Ilaria Roato, Francesca Sassi, Giuseppina Bisignano, Marta Saracco, Raffaele Pellerito, Salvatore Patanè, Riccardo Ferracini, Gian P Pescarmona, Giovanni C Isaia

**Affiliations:** 1 Gerontology Section, Department of Surgical and Medical Disciplines, University of Torino, Italy; 2 CeRMS (Center for Research and Medical Studies), A.O.U. San Giovanni Battista, Torino, Italy; 3 Division of rheumatology "Ordine Mauriziano" Hospital, Torino, Italy; 4 Department of Orthopaedics, A.O.U. San Giovanni Battista, Torino, Italy; 5 Department of Genetics, Biology and Biochemistry - University of Torino, Torino, Italy

## Abstract

**Background:**

Iloprost has been suggested to possess anti-inflammatory and immunomodulating actions and it is widely use as a vasodilatator in systemic sclerosis (SSc). In this study we evaluate the effect of iloprost on immune response in SSc patients. To this extend we enrolled 15 women affected by SSc and infused iloprost for 5 days. The effect of iloprost on T cells and monocytes was measured by flow cytometry, Real time PCR and measuring cytokines production in vivo and in vitro by ELISA.

**Results:**

Our results demonstrate that Iloprost reduces T cell and TNF alpha production both in vivo and in vitro. It reduces T regulatory cells number, but increases their activity after immune stimulation. It increases serum IL-2 and this increase persists 28 days after the last infusion, also RANKL was increased both in vivo and in vitro. We observed no effect on IFN gamma production.

**Conclusions:**

These results suggest that iloprost has anti-inflammatory and immunomodulating effects, reducing TNF alpha production by T cells and the number of T regulatory cells and increasing IL-2 and RANKL.

## Background

Systemic sclerosis (SSc) is a disease whose extremely complex, multifaceted pathogenesis cannot be referred to a single hypothesis, even if fundamental abnormalities in three or more types of cells are involved: fibroblasts, endothelial cells and cells of the immune system, namely T and B lymphocytes [1]. Mononuclear cells (mainly T cells) in the SSc skin infiltrates produce cytokines and growth factors responsible for the onset and progression of fibrosis and microvascular damage. The role of this immune dysfunction is not fully clear. Altered cellular immunity is revealed by aberrant T cell biology in both the skin, where the lesions display various features consistent with T cell activation [2-4], and the circulation, where CD4+ T cells are increased [2,5], and natural killer T cells are decreased [6]. Lastly, circulating T cells from SSc patients produce more inflammatory mediators compared to those from healthy controls [7,8].

An important role as controllers of self-reactivity [9] has recently been defined for T regulatory cells (Treg) in autoimmune disease. These cells are defined as CD4+/CD25^bright^/FoxP3+. In SSc, both an increase in their absolute number and a reduction of their function have been demonstrated [10].

A high proportion of SSc patients suffer from Raynaud's phenomenon. This is a vasospastic disorder that causes discoloration of the fingers, toes and occasionally other extremities, and its persistence may result in acral ulcer and significantly reduce the quality of life [11]. Prostaglandin (PG) analogues, particularly the PGI2 analogue iloprost, are widely used as vasodilators to treat this disorder [12].

Patients with SSc receiving Iloprost report a reduction in skin tightness, suggesting that this drug inhibits skin fibrosis. It has been suggested that iloprost reduces skin fibrosis by reducing collagen synthesis [13,14], the pro-fibrotic cytokine connective tissue growth factor [15] and the fibrotic response [16].

Iloprost has since been shown to possess anti-inflammatory and immunomodulating actions, both in vitro [17-20] and in vivo [21-24]. Of the two studies in humans [21,22], only Filaci et al. [22] evaluated the effect of iloprost in SSc. Its potential in this disease is thus an open question.

Here we demonstrate that iloprost decreases the production of TNF α by acting on T cells, ameliorates Treg function, and increases IL-2.

## Methods

### Patients

The study was approved by the "Clinical Study Review Committee" of Turin's "Ordine Mauriziano" Hospital, and all the patients signed an informed consent statement prior to their recruitment.

Fifteen women with SSc aged 53 ± 11 (7 in postmenopause) were enrolled. None were taking corticosteroids, estrogen, or immunosuppressants, or had previously received iloprost. Their SSc was classified as grade I accordng to Medsger et al. [17] and its mean duration was 26 ± 17 months. Raynaud's phenomenon was always present. All the patients complained of side-effects, but continued the infusions and reached satisfactory drug levels. The mean quantity infused was 0.8577 ± 0.1722 ng/Kg/min for 6 hrs/day for 5 days. All the patients started a second iloprost course after 28 days.

### Treatment

Iloprost was infused intravenously according to the standard protocol [11] for 6 h/day for 5 consecutive days. The target dose was 2 ng/kg/min.

Blood was drawn from an antecubital vein after an overnight fast of 10 or more hours at baseline, after the 5-day treatment, and then after a further 28 days to evaluate the timing of its action; 28 days were chosen since in clinical practice another course is started at the end of this period.

All measurements were performed from a single blood sample at a single time point per patient.

### Media, reagents, and chemicals

Cells were maintained in RPMI 1640 (Sigma-Aldrich, St Louis MO) supplemented with 2 mM L-glutamine 10% fetal bovine serum (FBS), benzyl penicillin (100 IU/ml) and streptomycin (100 μg/ml), phytohemoagglutinin (PHA: Sigma-Aldrich, St Louis MO.) was added in the stimulated condition.

IL-2 was purchased from R&D Systems (Minneapolis, MN, USA). Iloprost ([100 ng/ml], was kindly provided by Italfarmaco, Italy).

### Cells

Peripheral bood mononuclear cells (PBMCs) from SSc patients were obtained with the Ficoll-Paque method from 40 ml peripheral blood in lithium heparin at baseline, after 5 days iloprost infusion, and at 28 days after the last infusion. PBMC cultures were performed in triplicate for each subject in 96-well plates [1 × 10^5 ^cell/well]. For in vitro experiments PBMCs were obtained from buffy coats (five independent experiments).

### Ab

Fluorescein (FITC) conjugated anti-CD25, phycoerythrin (PE) conjugated anti-CD69 PE-conjugated anti-TNF α were purchased from Biolegend (San Diego, CA).

Peridinin chlorophyll protein (PeRCP) conjugated anti-CD3 was purchased from Becton & Dickinson (Bedford, MA)

T reg staining kit and APC-conjugated anti-CD14 were purchased from eBioscience (San Diego, CA).

### Flow cytometry

For intracellular staining, brefeldin ([10 μg/mL], Sigma-Aldrich, St. Louis, MO) was added to the cells for 1 hr. Cells were harvested, stained with surfaces antibodies, then permeabilized with saponin 5% (Sigma-Aldrich, St. Louis, MO) for 20 min washed and blocked for nonspecific binding sites using normal rat serum before the intracytoplasmic staining (TNF α and FoxP3) was performed. All the experiments were performed with cells stained with specific isotype control.

Flow cytometry was performed on a FACSCalibur flow cytometer (Becton Dickinson, Franklin Lakes, NJ, USA).

### Cytokine measurement

Serum IL-2 (Bender Med System, Vienna, Austria), total RANKL (BioVendor, madrice, Czech Repubblic), TNF α (DuoSet, R&D System Inc, Minneapolis, MN) and TGF β1 (DuoSet, R&D System Inc, Minneapolis, MN) were measured by ELISA. The levels of TNFα, free RANKL, TGF β1 and INFγ (Bender Med System, Vienna, Austria) were also measured in the supernatants from cultures stimulated or not with PHA.

### Real time RT-PCR

Total cellular RNA was isolated using TRIzol reagent (Ambion, Huntingdon, UK), chloroform extraction, and subsequent isopropanol precipitation according to the manufacturer's protocol. 1 μg of RNA was converted up to single-stranded cDNA by the High-Capacity cDNA Reverse Transcription Kit (Applied Biosystems, Warrington, UK). cDNA was stored at -20°C until use. β-Actin was the housekeeping control. RQ-PCR analysis of RANKL and TGFβ1 was performed by the iCycler iQ™ system (Bio Rad, Hercules, CA, USA). TaqMan probes were designed using Primer Express v2.0 software and synthesized by Applied Biosystems (Warrington, UK). FoxP3 expression was quantified by the Syber Green method. The following primers were used: RANKL sense 5'-GCCTTTTGCTCATCTCACTATTAATG-3' and antisense 5'-TGGTACCAAGAGGACAGACTCACTT-3'; TGF-b sense 5'- TTTGATGTCACCGGAGTTGTG-3' and antisense 5'- GCGAAAGCCCTCAATTTCC-3'; FOXP3, sense 5'-CAGCTGCTCGCACAGATTACTT-3' and antisense 5'-GGGACAGGATTGTGACATTTTGT-3'.

### Statistics

The normal distribution of each parameter was determined with Kurtosis' test: PBMC subsets, the cytokines (except for serum IL-2, serum RANKL and TGFβ in the supernatants) and genes measured were normally distributed. Student's paired t test was used to compare gaussian variables at baseline and after iloprost (5 and 28 days). Wilcoxon's test was used to compare non-gaussian variables at baseline and after iloprost (5 and 28 days).

The SPSS 17.0 software package was used to process the data with p < 0.05 as the significance cut-off

## Results

### Iloprost reduces T cell and TNF *α *production

Involvement of iloprost in control of the immune response and inflammation has been suggested [18-23], though there are no data indicative of this effect in SSc.

**Figure 1:**
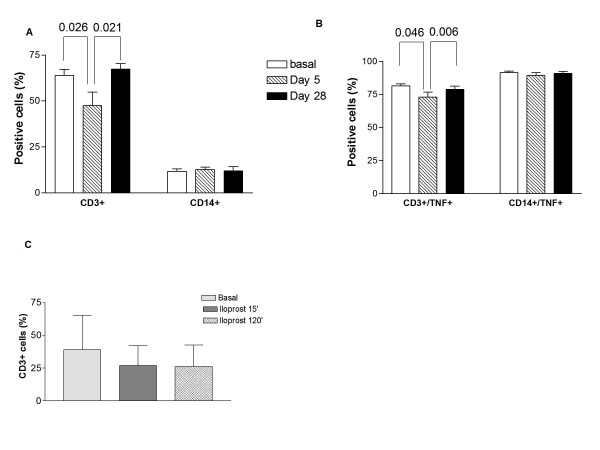
**Iloprost reduces T cells**. PBMCs isolated from SSc patients at baseline and after treatment with iloprost (5 days of daily treatment, 28 days after the last infusion) were analysed by flow cytometry to evaluate T cells, monocytes, TNF α production and degree of T cell activation. A. Graph shows the percentage of T cells (CD3+) and of monocytes (CD14+) among PBMCs. Bars represent mean and SD for all the patients, p values were calculated by a paired Student's t test, significant values are displayed. B. Graph shows the percentage of TNF α producing T cells (CD3+, gated on lymphocytes) and monocytes (CD14+, gated on monocytes). Bars represent mean and SD for all the patients, p values were calculated by a paired Student's t test, significant values are displayed. C. In vitro challenge of PBMCs culture with iloprost for 15 and 120 min. Graph shows the percentage of T cells (CD3+) in PBMCs. Bars represent mean and SD of five independent experiments. The reduction is not statistically significant.

1A illustrates the significant reduction of T cells achieved after 5 days of lloprost, whereas monocytes are not affected. T cells returned to the baseline level 28 days after the last infusion. As TNF α is produced mainly by T cells and monocytes among PBMCs and it is increased in inflammation, we looked at the effect of iloprost on TNF α producing cells. Figure 1B shows that TNF α producing T cells decreased after 5 days of iloprost and returned to baseline 28 days after the last infusion, whereas TNF α producing monocytes were not affected.

These findings could reflect diminished T cell activation, but evaluation of the early and late T cell activation markers CD69 and CD25 disclosed at non significant reduction of such activation (data not shown).

To determine whether iloprost reduces T cells directly, we incubated PBMCs from buffy coats (five experiments) with iloprost. Figure 1C suggests that iloprost might reduce the number of T cells in vitro, even though the results are not statistically significant.

**Figure 2:**
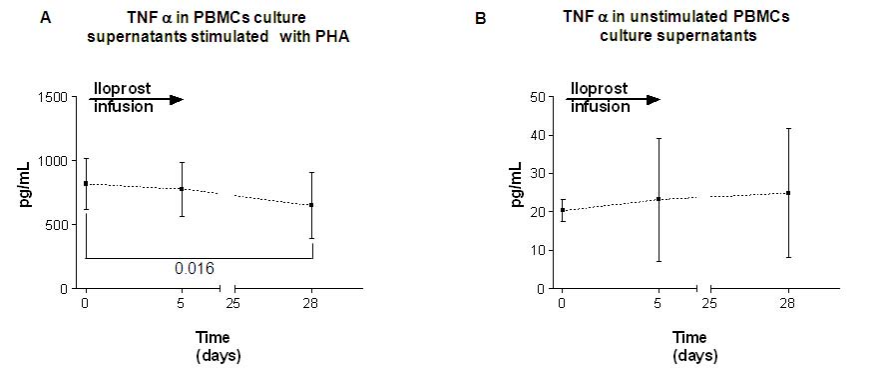
**Iloprost treatment reduces TNF α production from PBMCs after stimulus with PHA**. TNF α was measured in the culture supernatants of PBMCs from SSc patients treated with iloprost at baseline and after treatment (5 days of daily treatment, 28 days after the last infusion), cultures were stimulated or not with PHA which predominantly stimulates T cells. A. Graph shows the level of TNF α supernatants 24 hrs after stimulus with PHA. Symbols represent mean and SD for all the patients, p values were calculated by paired Students' T test, significant values are displayed. B. Graph shows the level of TNF α supernatants without PHA. Symbols represent mean and SD, for all the patients.

Confirmation of iloprost's anti TNF α effect was sought by measuring TNF α levels in supernatants from PBMC cultures stimulated or not with PHA. As expected, PHA increased TNF α secretion. Iloprost reduced this response and hence the production of TNF α (Figure 2A), whereas it had no effect on TNF α in the unstimulated culture (Figure 2B). We found no decrease in serum TNF α after iloprost (data not shown). This datum points to the specific action of iloprost against T cells and its ability to reduce TNF α production in response to T cell stimulation.

### Iloprost reduces T regs in vivo and in vitro

**Figure 3:**
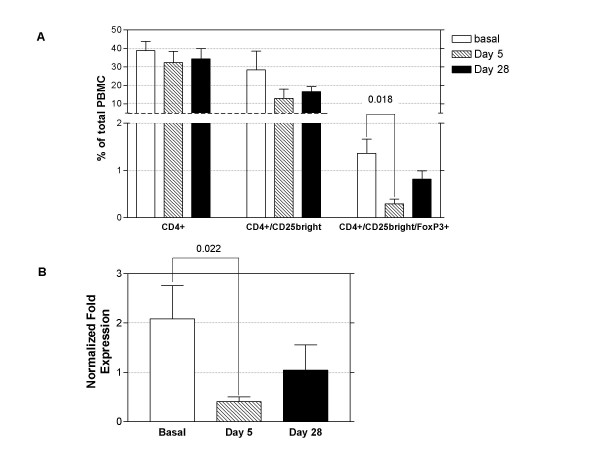
**Iloprost reduces Tregs**. PBMCs isolated from SSc patients at baseline and after treatment with iloprost (5 days of daily treatment, 28 days since the last infusion) were analysed by flow cytometry to evaluate Tregs (CD4+/CD25bright/FoxP3+ lymphocytes) and by real time PCR to evaluate FoxP3 expression. A. Graph shows the percentage of Tregs in PBMCs. Bars represent mean and SD for all the patients, p values were calculated with Student's paired t test, significant values are displayed. B. Graph shows FoxP3 expression in PBMCs from patients at baseline and after treatment. The FoxP3 expression was corrected for expression of the housekeeping gene β actin. Bars represent mean and SD for all the patients, p values were calculated with Student's paired t test, significant values are displayed.

Since altered Treg number and activity may be important in SSc [10], we evaluated these cells as a possible iloprost target. We studied T cells by flow cytometry and confirmed our data by analysing FoxP3 expression by real time RT-PCR. Figure 3A &3B demonstrate a reduction of Treg cells after 5 days of iloprost infusion.

**Figure 4:**
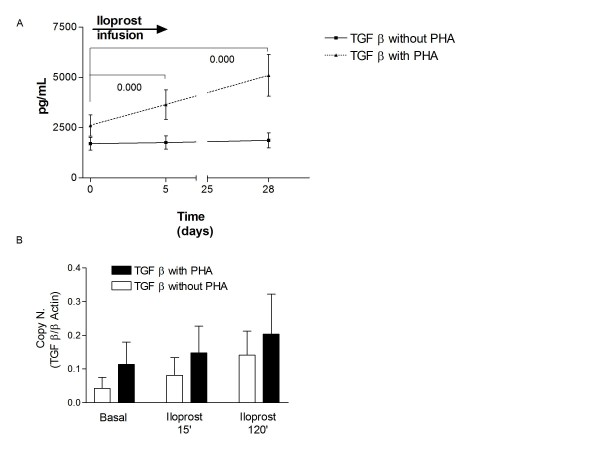
**Iloprost treatment increases TGF β production from PBMCs after stimulus with PHA**. TGF β was measured in the culture supernatants of PBMCs from SSc patients treated with iloprost at baseline and after treatment (5 days of daily treatment, 28 days after the last infusion), cultures were stimulated or not with PHA which predominantly stimulates T cells. A. Graph shows the level of TGF β supernatants 24 hrs after stimulus with PHA (dotted line) or without (continuous line). Symbols represent mean and SD, for all the patients, p values were calculated with the Wilcoxon test, significant values are displayed. B. In vitro challenge of PBMCs culture with iloprost for 15 and 120 min, after 24 hrs of PHA or without further stimulus. Graph shows the TGF β in PBMCs, TGF β expression was corrected for expression of the housekeeping gene β actin. Bars represent mean and SD of five independent experiments.

TGF β1 is mainly produced by Treg cells within PBMCs and it is a marker of their function [28,29], we measured this cytokine in the PBMCs culture supernatants at baseline and after iloprost with and without immune stimulation. The data in Figure 4A show a significant increase in TGF β1 in the PBMCs supernatants after stimulation with PHA following iloprost, hence suggest that iloprost influences the response of Treg cells to immune stimulation.

Serum TGF β 1 is not influenced by iloprost (data not shown): this datum points to a specific action of iloprost against T cells producing TGF β1, the Treg as TGFβ1 is produced by a variety of other cells in vivo.

Increased TGF β1 expression was also corroborated by real time PCR experiments on PBMCs incubated with iloprost for 15' and 120' in the presence or absence of PHA (Figure 4B).

Treg cells are mainly involved in maintenance of self-tolerance, which is disrupted in SSc as in other immune disorders [9,10,30,31]. Our data suggest that iloprost can enhance self tolerance and hence be useful in the treatment of SSc.

### Iloprost increases RANKL production

**Figure 5:**
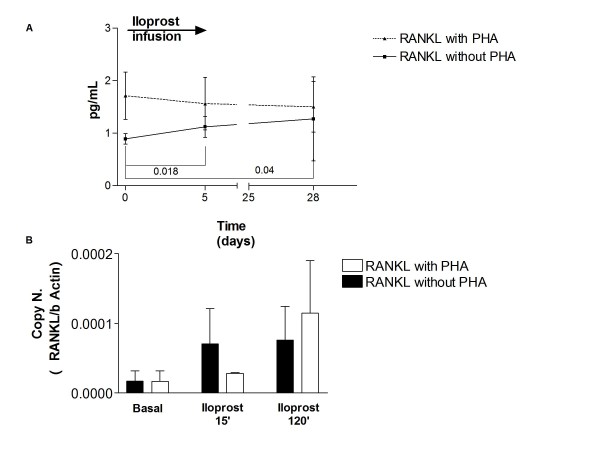
**Iloprost treatment increases RANKL production from PBMCs**. RANKL was measured in the culture supernatants of PBMCs from SSc patients treated with iloprost at baseline and after treatment (5 days of daily treatment, 28 days after the last infusion), cultures were stimulated or not with PHA which predominantly stimulates T cells. A. Graph shows the level of RANKL supernatants 24 hrs after stimulus with PHA (dotted line) or without (continuous line). Symbols represent mean and SD, for all the patients, p values were calculated with by Student's paired t test, significant values are displayed. B. In vitro challenge of PBMCs culture with iloprost for 15 and 120 min, after 24 hrs of PHA or without further stimulus. Graph shows the RANKL in PBMCs, RANKL expression was corrected for expression of the housekeeping gene β actin. Bars represent mean and SD of five independent experiments.

Receptor activator of nuclear factor κB ligand (RANKL) is a cytokine with pleiotropic functions deeply involved in control of the immune system, and increased in inflammation [24]. Among PBMCs it is mainly produced by T cells [33]. Here we investigated the effect of iloprost on this cytokine. Iloprost increases RANKL production by PBMCs in cultures both after 5 days of infusion and 28 days after the last infusion (Figure 5A), whereas it has no effect on serum RANKL (data not shown). In vitro PBMC stimulation with iloprost confirmed this in vivo effect (Figure 5B). These data agree with recent studies [34-36] in which RANKL increased following stimulus with PGE2. *Iloprost increases serum IL-2*.

IL-2 is a cytokine deeply involved in the control of T cell development and function [25], and controls Treg suppressor function [38]. Here we demonstrate that iloprost increases IL-2 levels of about 15% after 5 days (p = 0.008) and that this increase persists after the first course (about 40%, p = 0.007 vs baseline). To determine whether the effects of iloprost on TGFβ and RANKL production are mediated by IL-2, we incubated PBMCs from buffy coats with IL-2 and found that it had no effect on these cytokines (data not shown).

### Iloprost does not affect IFNγ levels

IFNγ stimulates the production of pro-inflammatory cytokines such as TNFα by macrophages [26]. It has a pleiotropic immune function [27], and may be involved in controlling T cell homeostasis [41, 42]. Iloprost does not affect serum and supernatant IFNγ (data not shown) in keeping with its lack of any effect on monocyte TNF production.

## Discussion

Prostaglandin I2 is a metabolite of arachidonic acid that has been shown to have anti-inflammatory functions [21,28,29], however, the cellular and molecular mechanisms of PGI2-mediated anti-inflammatory effects remain to be determined. Here we evaluated the effect of iloprost, a PGI2 analogue, widely used in the treatment of SSc, on T cells sub-populations and on pro-inflammatory cytokines.

This study shows that infusion of iloprost, according to the standard protocol to treat Raynaud's phenomenon in SSc patients, reduces T cells in the peripheral blood, especially TNF α producing T cells, according to previous in vitro [17-19] and in vivo [21] studies. The mechanism underling this effect was shown by Jorres et al. [19] that found that iloprost reduces TNF α transcriptional activity and decreases TNF α mRNA stability in human peripheral blood mononuclear leukocytes stimulated with LPS; Our study is the first evaluation of the effect of iloprost on TNF α production and T cell subsets in SSc. Our finding that iloprost acts on T cells only and not on monocytes is in contrast with Di Renzo et al.'s observation of a reduction of both monocytes and T cells [21]. This discrepancy may be attributable to the fact that they infused iloprost for 16 h/day for 7 days as required for critical limb ischemia. Our data show that iloprost reduces both the total amount of TNF α positive T cells and their ability to secrete TNF α after an antigenic stimulus. The role of TNF α in autoimmune diseases is well known, and it is currently a target for monoclonal antibody in diseases such as rheumatoid arthritis [30]. Its reduction by iloprost may thus be an important plus in the evaluation of therapeutic options.

Our study demonstrates a decrease in Treg after 5 days of iloprost infusion in vivo, associated with enhancement of their ability to produce TGF β1 in response to immune stimulation. This effect could be due to a generic immunomodulation by iloprost through a systemic increase in IL-2 production, as it is well known that this cytokine is deeply involved in the control of T cell development and function [25], and it controls Treg suppressor function [38]. Our study demonstrates that iloprost consistently increases serum IL-2 and that this increase persists 28 days after the last infusion. Here we show that IL 2 effect on TGF β1 production is not direct, but may be mediated trough other cell types in vivo. Recently a role for dendritic cells in the mediation of iloprost anti-inflammatory activity has been postulated [31,32]

The role of TFG β in determining the suppressor ability of Tregs is currently under debate [33], TGF β is crucial in determining the ability of Tregs to convert Foxp3- T cells into Foxp3+ T cells by a mechanism of infectious tolerance [34]. Recent data suggest an important role for Treg cells in the pathogenesis of autoimmune diseases [35], and it has been suggested that their number is increased, whereas their function is impaired, in SSc [10,30]. It may thus be supposed that the reduction in Treg number coupled with the increase in their TGF β production may be critical for SSc therapy.

We measured the levels of RANKL in serum and in supernatants because this molecule is important in inflammation and in the control of immune function [24] and expected they would be reduced after iloprost similarly to the reduction in TNF. After iloprost instead we found an increase in RANKL levels both as protein and as gene copies number. This could be interpreted as a class effect of prostaglandins, since recent studies [34-36] have demonstrated an increase in RANKL in cells after incubation with PGE2; moreover Conaway and colleagues demonstrated that both PGE2 and PGI2 were able to enhance osteoclast activity that is mainly under RANKL control [36]. However the RANKL increase is not due to IL-2 increase as demonstrated by the lack of IL-2 effect on in vitro PBMCs.

Our data demonstrated that iloprost does not influence IFN γ production. This cytokine is particularly involved in the inflammatory reaction of monocytes/macrophages and induces their TNF α secretion [26]; hence the lack of effect of iloprost on IFN is in line with the absence of its effect on monocyte TNF α production.

## Conclusions

This study suggests that iloprost has specific anti-inflammatory and immunomodulating effects: it reduces TNF α production by T cells without influence on TNF α monocyte production; it reduces the Tregs number, but ameliorates their function probably trough an increase in IL-2 and directly increases RANKL. Hence the therapeutic use of iloprost in SSc patients may be useful not only as vasodilator and antifibrotic, but also as immunomodulator agent.

## List of abbreviations

SSc: Systemic sclerosis; Treg: T regulatory cells; PG: Prostaglandin; PBMCs: peripheral blood mononuclear cells; PHA: phytohemoagglutinin; RANKL: Receptor activator of nuclear factor κB ligand

## Competing interests

The authors declare that they have no competing interests.

## Authors' contributions

PD designed the study and analysed the data. MAC, FS, GB performed the FACS analyses and the ELISA measurements. LD, LV, IR, SP performed the real time experiments MS, RP selected and treated the patients. All the authors critically revised the data, participate in the drafting of the manuscript and approved its final version.
